# Spatiotemporally specific roles of TLR4, TNF, and IL-17A in murine endotoxin-induced inflammation inferred from analysis of dynamic networks

**DOI:** 10.1186/s10020-021-00333-z

**Published:** 2021-06-24

**Authors:** Ruben Zamora, Sangeeta Chavan, Theodoros Zanos, Richard L. Simmons, Timothy R. Billiar, Yoram Vodovotz

**Affiliations:** 1 Department of Surgery, University of Pittsburgh, Starzl Biomedical Sciences Tower, 200 Lothrop St., Pittsburgh, PA 15213 USA; 2 Center for Inflammation and Regeneration Modeling, McGowan Institute for Regenerative Medicine, University of Pittsburgh, Pittsburgh, PA 15219 USA; 3 Pittsburgh Liver Research Center, University of Pittsburgh, Pittsburgh, PA 15261 USA; 4 Institute of Bioelectronic Medicine, The Feinstein Institutes for Medical Research, Manhasset, NY USA; 5 Center for Systems Immunology, University of Pittsburgh, Pittsburgh, PA 15213 USA

**Keywords:** Endotoxemia, Endotoxin, Lipopolysaccharide, TLR4, Cytokines, Chemokines, Dynamic Network Analysis, Principal Component Analysis

## Abstract

**Background:**

Bacterial lipopolysaccharide (LPS) induces a multi-organ, Toll-like receptor 4 (TLR4)-dependent acute inflammatory response.

**Methods:**

Using network analysis, we defined the spatiotemporal dynamics of 20, LPS-induced, protein-level inflammatory mediators over 0–48 h in the heart, gut, lung, liver, spleen, kidney, and systemic circulation, in both C57BL/6 (wild-type) and TLR4-null mice.

**Results:**

Dynamic Network Analysis suggested that inflammation in the heart is most dependent on TLR4, followed by the liver, kidney, plasma, gut, lung, and spleen, and raises the possibility of non-TLR4 LPS signaling pathways at defined time points in the gut, lung, and spleen. Insights from computational analyses suggest an early role for TLR4-dependent tumor necrosis factor in coordinating multiple signaling pathways in the heart, giving way to later interleukin-17A—possibly derived from pathogenic Th17 cells and effector/memory T cells—in the spleen and blood.

**Conclusions:**

We have derived novel, systems-level insights regarding the spatiotemporal evolution acute inflammation.

## Background

Inflammation is a protective response to microbial challenge or tissue injury and is regulated at multiple levels with positive and negative feedbacks (Systems et al. 2020; Kotas and Medzhitov 2015). Lipopolysaccharide (LPS) on the outer membrane of Gram-negative bacteria is a potent pathogen-associated molecular product (PAMP), stimulating a multi-system host response in the context of sepsis and septic shock (Opal 2010; Tan and Kagan 2014). Although an imperfect mimic of the dynamics of inflammation characteristic of true Gram-negative sepsis, LPS is widely used experimentally to stimulate acute inflammation (Parker and Watkins 2001).

Lipopolysaccharide interacts with multiple host soluble and cell-surface molecules, of which Toll-like Receptor 4 (TLR4) is the main receptor. Following binding to TLR4, LPS drives a cascade of events via MyD88 and TRIF, which ultimately regulate a multitude of inflammatory mediators in nearly all organs. The appearance of these inflammatory mediators in the systemic circulation is a hallmark of severe sepsis and related acute inflammatory states, and is commonly termed a “cytokine storm” (Tan and Kagan 2014).

We have begun to define the spatiotemporal evolution of LPS/TLR4-induced acute inflammation using a suite of computational approaches (Zamora 2018a). We utilized a variant of Principal Component Analysis (PCA) carried out over multiple time intervals (which we termed Time-Interval PCA [TI-PCA]) to identify organ-specific inflammatory trajectories over 0–48 h following intraperitoneal administration of LPS in mice (Zamora 2018a). This analysis suggested a spatiotemporal hierarchy in which the spleen appeared to be the initial site of inflammatory activation followed by the blood (plasma), heart, liver, and gut/lung. This analysis thus serves as an initial “roadmap” to the dynamics of inflammation in specific organs. In the present study, we sought to define the spatiotemporal cytokine/chemokine networks emerging from LPS/TLR4 interaction which take place simultaneously, or sequentially, within the various parenchymal organs, and that may be reflected in the systemic circulation.

## Methods

### Animals

All procedures involving animals complied with the regulations regarding the care and use of experimental animals published by the National Institutes of Health and were approved by the Institutional Animal Care and Use Committee of the University of Pittsburgh (Protocol No. 19014435). Male TLR4^+/+^ C57BL/6 mice were purchased from Jackson Laboratory (Bar Harbor, ME, USA). TLR4-null (TLR4^−/−^) mice were bred at the University of Pittsburgh animal facility on a C57BL/6 background (Deng 2013). Mice were allowed access to rodent chow and water ad libitum and used at the age of 8–12 weeks.

### Experimental procedures

Since numerous commercial LPS preparations contain measurable contaminating proteins, for this study we utilized ultra-purified LPS (from Escherichia coli O111:B4) purchased from List Biological Laboratories, Inc. (Campbell, CA). Mice (C57BL/6: n = 5–8 animals; TLR4^−/−^: n = 4 animals for each experimental group) were injected with LPS solution (3 mg/kg, i.p.) prepared in sterile PBS (control). At different time-points (0, 1, 4, 6, 12, 24 and 48 h), the animals were anesthetized with isoflurane (0.25–2% as needed), cardiac puncture was performed, blood was collected into heparinized tubes, and then centrifuged to obtain plasma; the mice were then euthanized by cervical dislocation while under anesthesia. Mice were then perfused with ice-cold PBS followed by RNALater™ (Thermo Fisher Scientific, Waltham, MA), which we have previously shown to be a preservation method compatible with Luminex™ analysis and equivalent to flash-freezing in liquid nitrogen (Barclay 2008). A small section (approx. 100 mg) of each tissue (liver [left lobe], heart, gut [terminal ileum], lung [left lobe], spleen, and kidney [left]) was collected and stored at −80 °C until analysis. Total protein isolation and determination was done as previously described (Metukuri 2009).

### Analysis of inflammatory mediators

Mouse inflammatory mediators were measured using a Luminex™ 100 IS apparatus (Luminex, Austin, TX) and the BioSource 20-plex™ mouse cytokine bead kit (BioSource-Invitrogen, San Diego, CA) as per manufacturer’s specifications. The antibody bead kit included: Granulocyte–Macrophage Colony-Stimulating Factor (**GM-CSF**), Interferon-γ (**IFN-γ**), Interleukin (**IL**)**-1α, IL-1β, IL-2, IL-4, IL-5, IL-6, IL-10, IL-12p40, IL-12p70, IL-13, IL-17A,** Interferon-γ-inducible Protein 10 (**IP-10/CXCL10**), Keratinocyte-derived Cytokine (**KC/CXCL1**), Monocyte Chemoattractant Protein-1 (**MCP-1/CCL2**), Monokine induced by Interferon-γ (**MIG/CXCL9**), Macrophage Inflammatory Protein-1α (**MIP-1α/CCL3**), Tumor Necrosis Factor**-**α (**TNF**), and Vascular Endothelial Growth Factor (**VEGF**). The final mediator concentrations are expressed in pg/ml for plasma samples, and in pg/mg total protein for tissue samples. Experimental data are presented as mean ± SEM.

### Machine learning analyses

*Dynamic Network Analysis (DyNA)* was carried out in order to define the central inflammatory network nodes as a function of both time and mouse strain. Using inflammatory mediator measurements of at least three time-points for experimental group, networks were created over five consecutive time periods (1–4 h, 4–6 h, 6–12 h, 12–24 h, and 24–48 h) using a modified version of our original algorithm implemented with MATLAB® software (Mi, et al. 2011; Ziraldo 2013; Abboud 2016). Network edges/connections (or trajectories of inflammatory mediators that move in parallel; positive: same direction; negative: opposite direction) were created if the Pearson correlation coefficient between any two nodes (inflammatory mediators) at the same time-interval was 1) greater or equal to an absolute value threshold of 0.7 and 2) different from Control (0 h), as indicated. The network complexity for each time-interval was calculated using the following formula: Sum (N_1_ + N_2_ + … + N_n_)/n−1, where N represents the number of connections for each mediator and n is the total number of mediators analyzed. The total number of connections represents the sum of the number of connections across all time-intervals for all mice in each sub-group.

*Spearman’s correlation* was carried out to measure the strength of the association between the Luminex™ data for two different mediators using a modified version of a MATLAB® -based toolbox described previously (Pernet et al. 2012).

## Results

### Dynamic Network Analysis suggests a hierarchy of organ-specific, TLR4-dependent response to LPS and supports a role for TNF-driven inflammation in the hearts of C57BL/6 mice

**Fig. 1:**
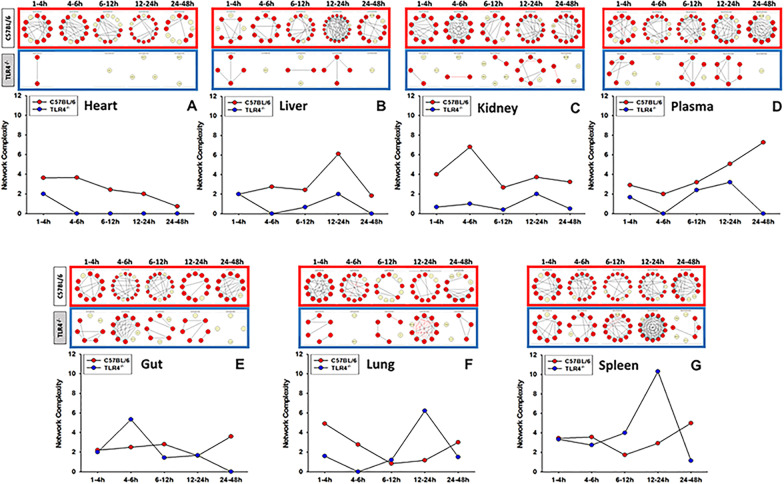
Dynamic Network Analysis of inflammatory mediators shows different network complexity in C57BL/6 and TLR4^−/−^ mice. Animals were injected with LPS (3 mg/kg, i.p.). At different time points upon sacrifice, blood and different organs (liver, heart, gut, lung, spleen and kidney) were collected, inflammatory mediators were measured by Luminex™ and DyNA (stringency level = 0.7) was performed during each of the following five time frames: 1–4 h, 4–6 h, 6–12 h, 12–24 h, and 24–48 h as described in Section “Materials and methods”. Figure shows an overview of all the individual networks and mediator connections in both C57BL/6 and TLR4^−/−^ mice during each time-frame for heart (Panel **A**), liver (Panel **B**), kidney (Panel **C**) plasma (Panel **D**), gut (Panel **E**), lung (Panel **F**), and spleen (Panel **G**). Circles in yellow represent mediators that changed significantly compared to baseline (0 h) but had no connections to other mediators. Red circles represent mediators that changed significantly compared to baseline and are connected to other mediators. Black and red arrows represent positive and negative connections, respectively

As shown previously (Zamora 2018b), the significant reduction in systemic inflammation in TLR4^−/−^ mice was associated with lower circulating concentrations of ALT, reflective of a lesser degree of organ (predominantly liver) damage in TLR4^−/−^ mice. We first sought to define the TLR4-dependent spatiotemporal evolution of LPS-induced inflammatory networks. DyNA (Mi, et al. 2011; Ziraldo 2013; Zamora, et al. 2016) was employed to define and compare the interconnections among inflammatory mediators in C57BL/6 vs. TLR4^−/−^ mice over five defined time-intervals (1–4 h, 4–6 h, 6–12 h, 12–24 h, and 24–48 h). This analysis showed differential dynamic inflammation networks in C57BL/6 as compared to TLR4^−/−^ mice (Fig. 1). DyNA also suggested a hierarchy of TLR4 sensitivity in the response to LPS in various organs: inflammation in the heart appeared to be the most dependent on TLR4 (Fig. 3A), followed by the liver (Fig. 1B), kidney (Fig. 1C), plasma (Fig. 1D), gut (Fig. 1E), lung (Fig. 1F), and spleen (Fig. 1G). Higher dynamic network connectivity in the gut, lung, and spleen of TLR4^−/−^ mice vs. C57BL/6, especially in the 12–24 h time frame in lung and spleen (Figs. 1F–G) raises the possibility of non-TLR4 LPS signaling pathways in those organs.

**Fig. 2:**
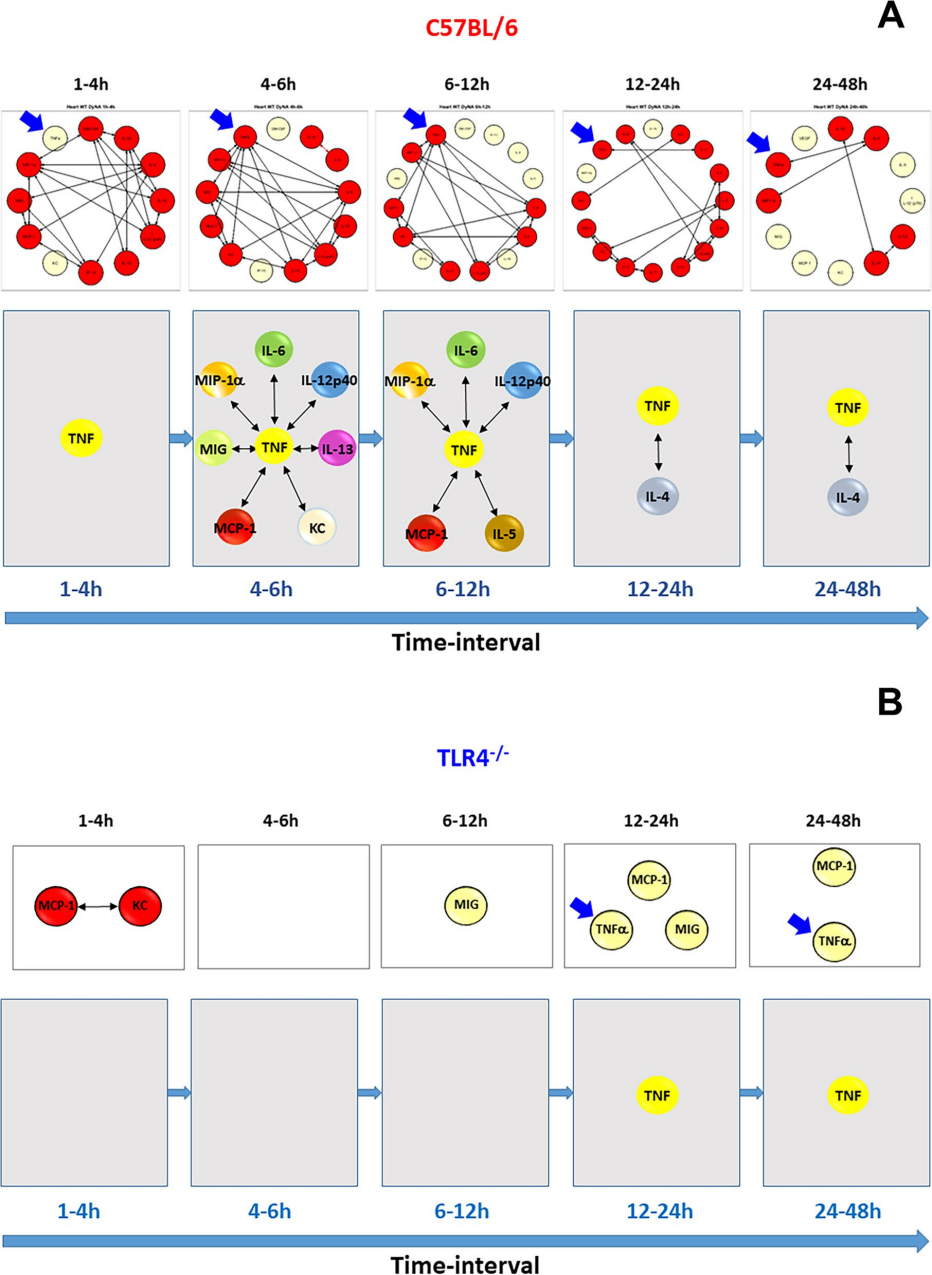
Differential Dynamic Network Analysis of inflammatory mediators in the heart of C57BL/6 and TLR4^−/−^ mice. Animals were injected with LPS (3 mg/kg, i.p.). At different time points upon sacrifice the heart was collected and inflammatory mediators were measured by Luminex™. DyNA (stringency level = 0.7) was then performed during each of the following five time-frames: 1–4 h, 4–6 h, 6–12 h, 12–24 h, and 24–48 h as described in *Materials and Methods*. Figure shows an overview of all the individual networks and mediator connections in the heart of C57BL/6 mice (**A**, top row) and the detailed connectivity for TNF (blue arrows) during each of the five time-frames analyzed (**A**, bottom row). The same analysis for the heart of TLR4^−/−^ mice is shown in (**B**)

Based on this initial analysis, we next hypothesized the presence of a TLR4-dependent, TNF-centered inflammatory network from 4 to 12 h in the hearts of C57BL/6 mice. In support of this hypothesis, DyNA suggested that TNF is among the most connected mediators in the heart in the 4–12 h time interval in C57BL/6 mice (Fig. 2A) but plays no such role in TLR4^−/−^ mice (Fig. 2B). It is important to note that during this time interval TNF was not among the dominant mediators in the C57BL/6 heart as determined by TI-PCA previously (Zamora 2018a), but DyNA suggested that TNF plays a central role in the inflammatory network during 4–12 h (Fig. 2A). Notably, DyNA also suggested a significant connectivity of TNF to MIP-1α, IL-5, IL-6, and IL-12p40 (Fig. 2A), mediators shown to be dominant by TI-PCA (Zamora 2018a).

**Fig. 3:**
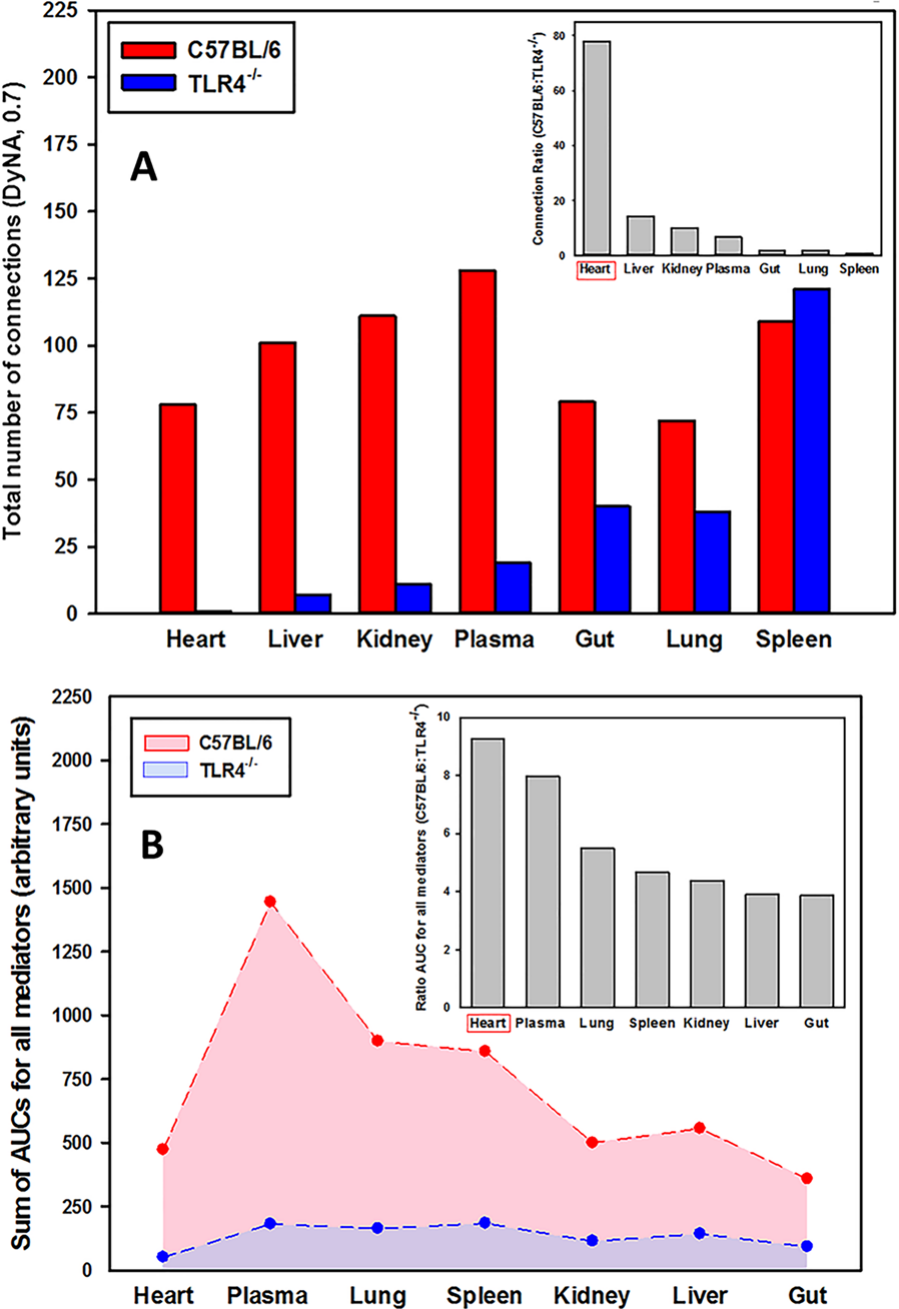
Dynamic Network Analysis of inflammatory mediators shows different inflammatory connectivity in C57BL/6 vs. TLR4^−/−^ mice. Animals were injected with LPS (3 mg/kg, i.p.). At different time points upon sacrifice, blood and different organs (liver, heart, gut, lung, spleen and kidney) were collected, inflammatory mediators were measured by Luminex™ and DyNA (stringency level = 0.7) was performed during each of the following five time frames: 1–4 h, 4–6 h, 6–12 h, 12–24 h, and 24–48 h as described in *Materials and Methods*. **A** shows the total number of connections for plasma and different organs. The mediator connection ratio (C57BL/6:TLR4^−/−^) calculated from the total number of connections is shown in the inset. **B** shows the sum of AUCs for all mediators calculated over 0–48 h in heart, plasma, lung, spleen, kidney, liver and gut. The inset depicts the ratio of AUCs (C57BL/6:TLR4^−/−^) for plasma and different organs calculated from the sum of AUCs for all mediators shown in the main figure

When comparing the total number of DyNA connections in all organs, this analysis also showed a significantly higher overall connectivity in C57BL/6 vs. TLR4^−/−^ (Fig. 3A) in all organs except spleen, with the heart as the organ with the greatest fold difference in total number of connections (Fig. 3A, inset). In addition, the sum of AUCs for all mediators for each organ clearly differentiated C57BL/6 mice (higher AUCs) from TLR4^−/−^ mice (lower AUCs) (Fig. 3B), with the heart being the organ with the highest ratio of sum of AUCs for all mediators in C57BL/6 vs. TLR4^−/−^ (Fig. 3B, inset).

**Fig. 4:**
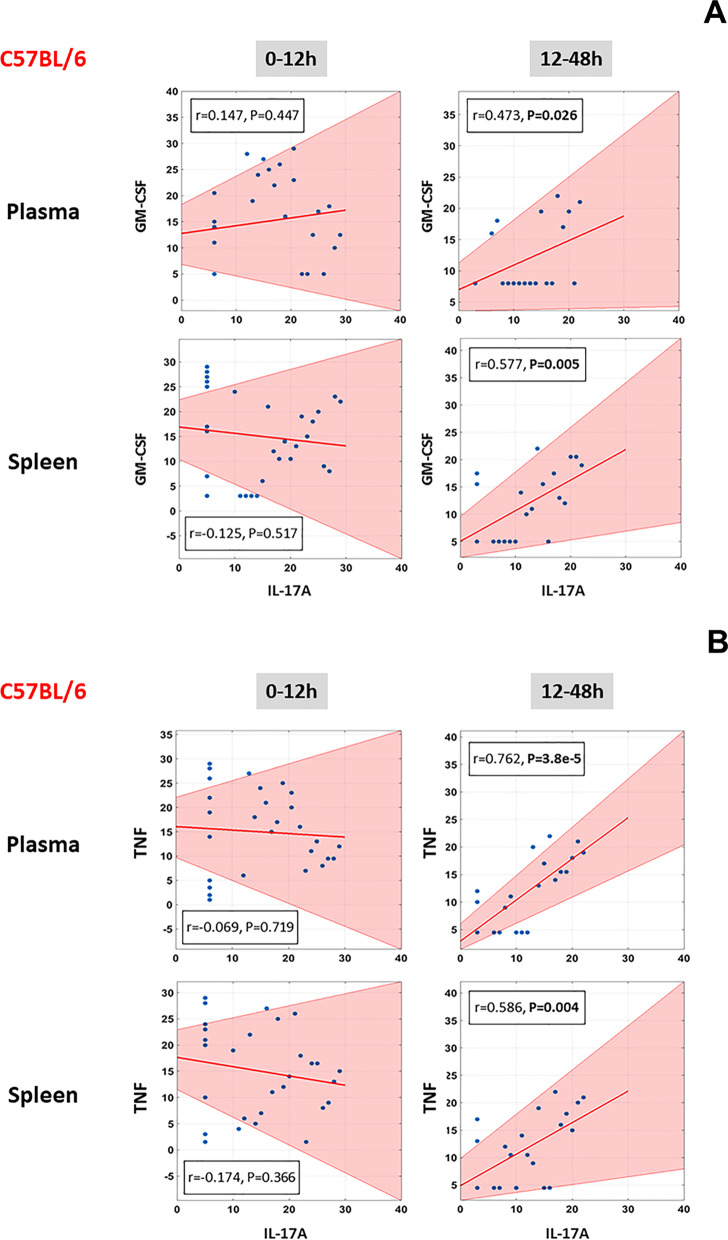
Correlation between levels of IL-17A/GM-CSF and IL-17A/TNF in plasma and spleen of C57BL/6 mice. Animals were injected with LPS (3 mg/kg, i.p.). At different time points upon sacrifice, the inflammatory mediators in blood and spleen were measured by Luminex™ as described in *Materials and Methods*. The plots show the Spearman’s correlations between levels of IL-17A/GM-CSF (**A**) and IL-17A/TNF (**B**) during the 0–12 h and 12–48 h time-intervals (the shaded area represents the 95% bootstrapped confidence interval)

**Fig. 5:**
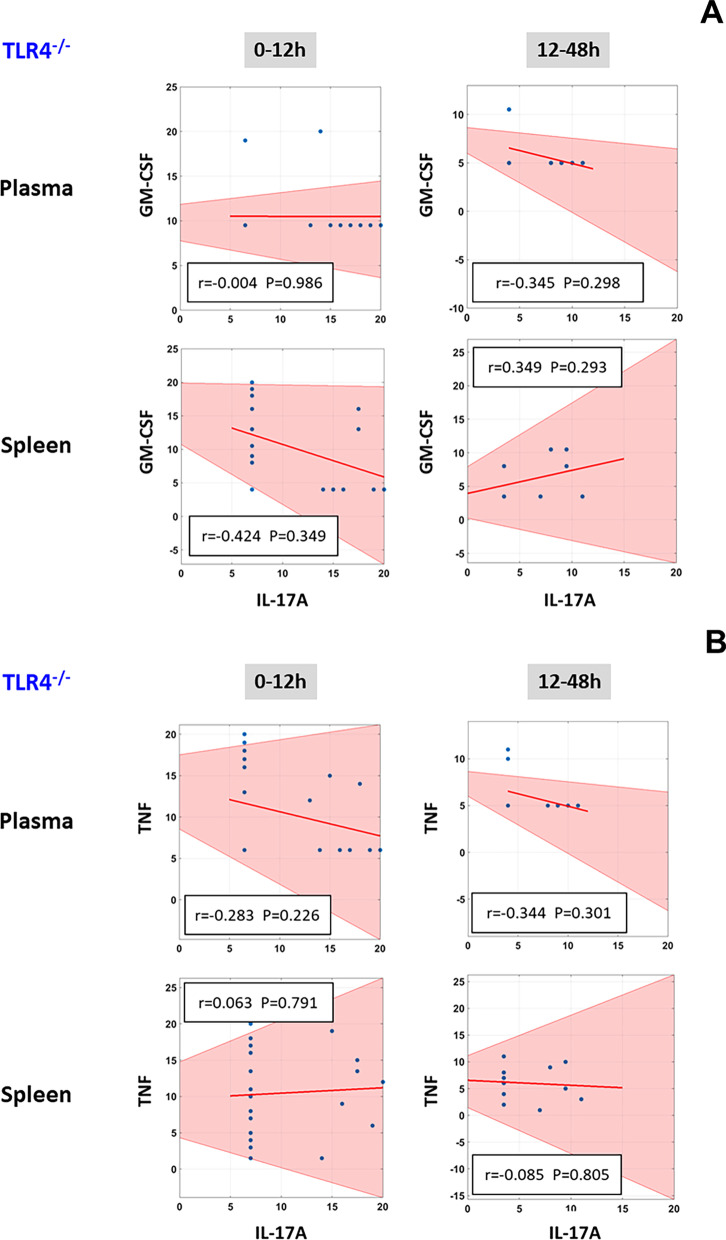
Correlation between levels of IL-17A/GM-CSF and IL-17A/TNF in plasma and spleen of TLR4^−/−^ mice. Animals were injected with LPS (3 mg/kg, i.p.). At different time points upon sacrifice, the inflammatory mediators in blood and spleen were measured by Luminex™ as described in *Materials and Methods*. The plots show the Spearman’s correlations between levels of IL-17A/GM-CSF (**A**) and IL-17A/TNF (**B**) during the 0–12 h and 12–48 h time-intervals (the shaded area represents the 95% bootstrapped confidence interval)

Taken together, these results demonstrate (1) highly distinct inflammation network complexity trajectories across organs, and further differences between C57BL/6 and TLR4^−/−^ mice (Fig. 1); (2) a spike in complexity between 12 and 24 h in the liver of C57BL/6 mice and a higher initial complexity that decreased to similar levels after 4 h in the liver of TLR4^−/−^ mice (Fig. 1B); (3) increasing dynamic network complexity in plasma of C57BL/6 mice after 6 h (Fig. 1D); (4) a spike in complexity from 4 to 6 h in the terminal ileum [gut] of TLR4^−/−^ mice (Fig. 1E); (5) a spike in complexity between 12 and 24 h with many negative connections in the lung of TLR4^−/−^ mice (Fig. 1F); and (6) a spike in complexity between 12 and 24 h with many positive connections in the spleen of TLR4^−/−^ mice (Fig. 1G).

### Correlations between levels of IL-17A and GM-CSF/TNF in C57BL/6 mice suggest a role for pathogenic Th17 cells and effector/memory T cells in LPS/TLR4-induced inflammation

In our prior studies, TI-PCA suggested that in the 12-48 h time-interval the inflammatory response was mostly dominated by IL-17A in C57BL/6 mice but not in TLR4^−/−^ animals (Zamora 2018a). The cytokine IL-17A can be produced by a variety of cell types, including Th17 cells, innate lymphoid cells, γδ T cells, and both CD4^+^ and CD8^+^ effector/memory T cells (Weaver et al. 2007; Korn et al. 2009; Cua and Tato 2010). Kuchroo and co-workers have described a sub-population of Th17 cells known as pathogenic Th17 cells, which are implicated in driving pathological inflammatory processes; these cells, in addition to expressing IL-17A, are also characterized by their expression of GM-CSF (Peters et al. 2011). We therefore hypothesized that the signature of pathogenic Th17 cells would be present in the 12–48 h time interval in plasma and the spleen. As shown in Fig. 4A, at the 12–48 h time-interval (but not earlier) we found a significant positive correlation between the levels of those two mediators in both plasma and spleen of C57BL/6 mice (Fig. 4A), but not in TLR4^−/−^ animals (Fig. 5A).

Recent studies report that CD4^+^/CD8^+^ effector/memory T cells express IL-17A along with TNF (Langrish 2005; McArthur and Sztein 2013). At the 12-48 h time-interval (but not earlier), there was a significant positive correlation between the levels of IL-17A and TNF in both plasma and spleen of C57BL/6 mice (Fig. 4B), but not in TLR4^−/−^ animals (Fig. 5B), suggesting the possible presence of this cell type as well.

## Discussion

We have previously documented the spatiotemporal spread of inflammatory programs across multiple organs based on an approach involving over 10,000 data points on the dynamics of inflammatory mediators in multiple organs at the protein level combined with data-driven computational modeling. Those studies also defined a time frame after which organ-localized inflammation appeared in the systemic circulation (a major hallmark of a pathological “cytokine storm”) (Zamora 2018a). We expanded this analysis in the present study to define dynamic networks of inflammation in individual organs and the role of TLR4 therein. Unexpectedly, we inferred a role for the heart in propagating multi-organ inflammation, as well as obtaining novel insight regarding organ-specific roles of key inflammatory cells (pathogenic and memory/effector T cells) and cytokines (TNA and IL-17A).

We focused on endotoxemia as a quantitative, reproducible experimental model of acute inflammation, with hallmarks of both sepsis and sterile inflammation (Parker and Watkins 2001). We confirmed and extended many findings regarding the effects of LPS and the role of TLR4 on various organs: based on DyNA, we inferred a higher multi-compartment network density in C57BL/6 vs. TLR4^−/−^ mice, thereby adding nuance to the concept of generally lower inflammatory response to LPS in the absence of TLR4 (Beutler 2000,2002; Beutler and Rietschel 2003).

Our computational network analyses represent an overarching hypothesis about how the inflammatory response progresses. In this hypothesis, both parenchymal and inflammatory cells (resident and infiltrating) sense the presence of LPS and, in response, elaborate chemokines that form defined networks. As the presence of signals regarding the original stress (in the form of LPS), along with the development and actions of these chemokine networks, early regulatory cytokines such as TNF begin to be secreted. Due to their dependence on the ongoing dynamic flow of information through chemokine networks at early time points, these mediators are present at low levels, often with high variance, and thus may be considered “insignificant” using standard statistical analyses. However, their presence and effect may be inferred using computational techniques such as PCA or DyNA (Namas 2015). Future studies will examine the potential crosstalk among dynamic networks to add nuance and detail to this emerging dynamic picture.

Multiple prior studies had documented TLR4-dependent effects on the liver (Bigorgne 2016; Ouyang 2016), lung (Guillot 2004) and kidney (Cunningham et al. 2004). In the current study, DyNA focused our attention on the heart, suggesting that TNF connectivity in the hearts of mice expressing TLR4 peaked at the same time intervals as those predicted by TI-PCA in our prior study (Zamora 2018a). Prior studies suggested that LPS-induced inflammation impacts the hearts of C57BL/6 mice. For example, exposure to LPS was associated with reduced cardiac function, increased myocardial levels of IL-1β and TNF, and upregulation of TLR4 mRNA prior to myocardial leukocyte infiltration, suggesting an impact on local parenchymal and/or resident inflammatory cells; these effects were absent in TLR4^−/−^ mice (Fallach 2010). In other studies, the expression of TNF, IL-1α, IL-2, IL-4, IL-5, IL-6, IL-10, IL-17A, IFN-γ, and GM-CSF was lower in the infarcted area of the hearts of TLR4^−/−^ mice vs. wild-type (Timmers 2008). Herein, we speculate that the heart is involved not only as an end-target but also in the propagation of multi-organ inflammation induced by LPS. This is supported not only by our prior (Zamora 2018a) and current studies, but also by studies in experimental murine hemorrhagic shock (Meldrum 1997). Further studies are needed to test this intriguing hypothesis.

The role of the heart in propagating inflammation may be tied to the cytokine IL-17A. Our studies using TI-PCA implicated IL-17A in LPS/TLR4-induced inflammation in the heart at 4–6 h following LPS administration; in the heart, spleen, liver, kidney, and gut at 6–12 h; in the liver, kidney, spleen, gut, and plasma at 12–24 h; and in the heart, lung, and plasma at 24–48 h (Zamora 2018a). The discovery of the key roles of IL-17A and IL-17A-producing cells in inflammation, autoimmune diseases, and host defense has led to the experimental targeting of the IL-17A pathway in different animal models as well as in clinical trials in humans (Flierl 2008; Freitas 2009; Chen and Kolls 2017). Th17 cells that differ in their inflammatory potential have been described, including IL-10-producing Th17 cells that are weak inducers of inflammation; highly inflammatory, IL-23-driven, GM-CSF/IFN-γ-producing Th17 cells (Kara 2015); and CD4^+^/CD8^+^ effector/memory T cells (Mills 2008). Furthermore, apart from circulating T cells or those from lymphoid sites, large numbers of T cells reside in multiple peripheral tissue sites including lung, intestine, liver, and skin as non-circulating, tissue-resident memory T cells (termed Trm cells) (Turner et al. 2014). Notably, recent studies suggest the presence of Th17 cells in autoimmune myocarditis (Hua 2020). In the present study, we observed a significant positive correlation between IL-17A and GM-CSF, cytokines secreted by these pathogenic Th17 cells, as well as a correlation between IL-17A and TNF, which have been suggested as hallmarks of CD4^+^/CD8^+^ effector/memory T cells residing in the spleen. These results may indicate a broad-based, TLR4-dependent deficiency in the differentiation of these cell sub-populations. In support of this hypothesis, LPS is known to drive Th17 differentiation directly (Park et al. 2015), and this Th17 differentiation requires TLR4 (Qu 2019). These findings may have clinical implications, since another study suggests that LPS promotes a Th17 bias in patients with Non-alcoholic Steatohepatitis (NASH) (Wang 2020). Notably, inhibition of IL-17A protected mice from both experimental sepsis (Flierl 2008) and trauma/hemorrhage (Abboud 2016), consistent with a central organizing role of IL-17A in self-sustaining inflammatory networks (Abboud 2016). Future work will aim to identify (and quantify) the presence and activity of specific inflammatory or parenchymal cells in the heart and other organs during specific time periods, including studies aimed at a more precise identification of the specific cell types that produce IL-17A in this system.

While TLR4 deficiency is associated with the abrogation of most of the canonical responses to LPS (Beutler 2000,2002; Beutler and Rietschel 2003), recent studies have suggested that the caspase-11 pathway may also be involved in LPS signaling (Hagar et al. 2013; Kayagaki 2013). Our findings of elevated but negative network connectivity in the lung even in the absence of TLR4 suggest a role for other LPS signaling pathways such as caspase 11, which need to be investigated further.

Several compartments were not interrogated in our study, and this is a limitation. For example, the peritoneal cavity (interrogated via lavage) exhibited increased levels of TNF, IFN-γ, and IL-10 following LPS stimulation for 2 h (Matalka et al. 2005). A major TNF response has been described in fat-associated lymphoid clusters after exposure to LPS (Benezech 2015). We did not study these compartments, nor did we interrogate skin, muscle, bone, interstitium, or circulating leukocytes.

Finally, increased levels of TNF, IFN-γ and IL-10 were detected in brain tissue within 2 h of exposure to LPS (Matalka et al. 2005), which suggests that the central role of the brain in regulating LPS-induced acute inflammation (Tracey 2002; Thomson et al. 2014) also must be integrated into the emerging picture presented in the present study. One of the main conduits of this neuro-immune communication is the vagus nerve. Stimulation of the vagus nerve attenuates cytokine production and improves survival in experimental sepsis, hemorrhagic shock, ischemia–reperfusion injury, and other conditions of cytokine excess (Johnston and Webster 2009; Das 2011; Chavan et al. 2017; Chavan and Tracey 2017). Moreover, the vagus also carries afferent signals that communicate both LPS exposure, as well as cytokine specific information to the brain, in the form of TNF or IL-1β signaling (Steinberg 2016; Zanos 2018). Nevertheless, the dynamics of both afferent and efferent vagus signaling, as they relate organ specific inflammation are not yet thoroughly understood, leaving their causal relationship to the dynamics described in this study an important open question.

In summary, we have used computational modeling to further delineate the complex spatiotemporal dynamics of LPS/TLR4-induced inflammation. This methodology may be useful for studying other complex, dynamic, multi-compartment biological systems in vivo.

## Data Availability

The datasets used and/or analyzed during the current study are available from the first and corresponding authors on reasonable request.

## Abbreviations

LPS: Lipopolysaccharide
GM-CSF: Granulocyte–Macrophage Colony Stimulating Factor
IFN: Interferon
IL: Interleukin
IP-10: IFN-γ-inducible Protein of 10 kDa/CXCL10
KC: Keratinocyte-derived Cytokine/CXCL1
MCP-1: Monocyte Chemotactic Protein-1/CCL2
MIG: Monokine induced by γ-interferon/CXCL9
MIP-1α: Macrophage Inflammatory Protein-1α (MIP-1α/CCL3α)
TNF: Tumor Necrosis Factor-α
VEGF: Vascular Endothelial Growth Factor
PCA: Principal Component Analysis
TI-PCA: Time-Interval Principal Component Analysis
DyNA: Dynamic Network Analysis
